# The Role of Structural Maintenance of Chromosomes Complexes in Meiosis and Genome Maintenance: Translating Biomedical and Model Plant Research Into Crop Breeding Opportunities

**DOI:** 10.3389/fpls.2021.659558

**Published:** 2021-03-31

**Authors:** Pablo Bolaños-Villegas

**Affiliations:** ^1^Fabio Baudrit Agricultural Research Station, University of Costa Rica, Alajuela, Costa Rica; ^2^Lankester Botanical Garden, University of Costa Rica, Cartago, Costa Rica

**Keywords:** meiosis, structural maintenance of chromosomes, cohesin, DNA recombination, food security, stress tolerance

## Abstract

Cohesin is a multi-unit protein complex from the structural maintenance of chromosomes (SMC) family, required for holding sister chromatids together during mitosis and meiosis. In yeast, the cohesin complex entraps sister DNAs within tripartite rings created by pairwise interactions between the central ring units SMC1 and SMC3 and subunits such as the α-kleisin SCC1 (REC8/SYN1 in meiosis). The complex is an indispensable regulator of meiotic recombination in eukaryotes. In *Arabidopsis* and maize, the SMC1/SMC3 heterodimer is a key determinant of meiosis. In *Arabidopsis*, several kleisin proteins are also essential: SYN1/REC8 is meiosis-specific and is essential for double-strand break repair, whereas AtSCC2 is a subunit of the cohesin SCC2/SCC4 loading complex that is important for synapsis and segregation. Other important meiotic subunits are the cohesin EXTRA SPINDLE POLES (AESP1) separase, the acetylase ESTABLISHMENT OF COHESION 1/CHROMOSOME TRANSMISSION FIDELITY 7 (ECO1/CTF7), the cohesion release factor WINGS APART-LIKE PROTEIN 1 (WAPL) in *Arabidopsis* (AtWAPL1/AtWAPL2), and the WAPL antagonist AtSWITCH1/DYAD (AtSWI1). Other important complexes are the SMC5/SMC6 complex, which is required for homologous DNA recombination during the S-phase and for proper meiotic synapsis, and the condensin complexes, featuring SMC2/SMC4 that regulate proper clustering of rDNA arrays during interphase. Meiotic recombination is the key to enrich desirable traits in commercial plant breeding. In this review, I highlight critical advances in understanding plant chromatid cohesion in the model plant *Arabidopsis* and crop plants and suggest how manipulation of crossover formation during meiosis, somatic DNA repair and chromosome folding may facilitate transmission of desirable alleles, tolerance to radiation, and enhanced transcription of alleles that regulate sexual development. I hope that these findings highlight opportunities for crop breeding.

## Introduction

Our species faces a vexing problem. In the past, agricultural production kept pace with a growing population. However, by the year 2050, crop production will no longer meet demand. Increased consumption of meat and dairy in the developing world is worsened by biofuel production, depletion of water resources, and global warming. Thus, breeding of new varieties is key to boosting yields and matching future demand (Scheben and Edwards, 2018).

Global warming is often mentioned because of its negative effect on rainfall, but anthropogenic climate change is also believed to progressively suppress cloud formation, thus increasing exposure to UVB radiation (320–290 nm; Diffey, 2002; Lindfors and Arola, 2008; Schneider et al., 2019). The Food and Agriculture Organization of the United Nations has well-documented the reduced crop yield and total dry weight resulting from enhanced exposure to UVB radiation in the field for crops such as rice (*Oryza sativa* L., Poaceae), soybean (*Glycine max* L., Fabaceae), corn (*Zea mays* L., Poaceae), potatoes (*Solanum tuberosum* L., Solanaceae), pea (*Pisum sativum* L., Fabaceae), and sugar cane (*Saccharum officinarum* L., Poaceae; Krupa and Jäger, 2006). Exposure to a high dose of UVB radiation in C3 and C4 species exacerbates sensitivity to drought by reducing leaf conductance, water use efficiency, and leaf area. It also decreases floral yield, fruit set, and fruit yield (Krupa and Jäger, 2006). When combined with increased atmospheric CO_2_, UVB radiation worsens crop performance (Wijewardana et al., 2016). Depletion of atmospheric ozone (O_3_) and the anthropogenic release of atmospheric aerosols such as nitrogen oxides can also alter surface UV flux by their distribution in the atmospheric column (Erickson et al., 2015), which adds a layer of complexity to an already difficult scenario of climate instability.

In plants, the two major products of UVB damage are cyclobutene pyrimidine dimers and pyrimidine pyrimidone photoproducts, which are mostly repaired by cyclobutene pyrimidine dimers and 6–4 photolyases (Sakamoto, 2019). Also, extensive evidence indicates that nucleotide excision repair plays an important role in removing damage caused by UV radiation (Nisa et al., 2019; Sakamoto, 2019). However, UV radiation is also toxic because it distorts the template structure of DNA and prevents replication (Kim et al., 2019; Sakamoto, 2019). In turn, these stalled replication sites create fragile single-strand regions that easily lead to highly toxic double-strand breaks (DSBs; Nisa et al., 2019; Sakamoto, 2019). DSBs are repaired by translesion synthesis with the help of a translesion synthesis polymerase or by template switch and homologous recombination. The latter process relies on PROLIFERATING CELL NUCLEAR ANTIGEN (PCNA) polyubiquitination, the combined action of RAD51 and BRCA1 (Kim et al., 2019; Nisa et al., 2019; Sakamoto, 2019) and activity of the cohesin complex (Bolaños-Villegas et al., 2013).

Another serious genotoxic stress that affects output in crops such as rice and barley is the soil content of aluminum (Al; Awasthi et al., 2017; Jaskowiak et al., 2018), especially in acidic soils such as in India (Awasthi et al., 2017). In such soils (pH < 5.5), Al solubilizes into the phytotoxic species Al^3+^ from non-toxic silicates and oxides (Awasthi et al., 2017; Sjögren and Larsen, 2017). In flooded fields, the presence of Al^3+^ leads to an increase in soluble Fe^2+^ content, also leading to iron toxicity (Awasthi et al., 2017). The most severe symptoms of Al toxicity are rapid inhibition of root elongation caused by damage in cells at the root apex and reduction in crop yield by 20–40% (Panhwar et al., 2015; Awasthi et al., 2017). In barley roots, exposure to Al^3+^ causes the formation of micronuclei and apoptosis (Jaskowiak et al., 2018). Flow cytometry also revealed a delay in cell division, as evidenced by an increase in frequency of cells in the G2/M phase (Jaskowiak et al., 2018). Exposure to Al^3+^ in *Arabidopsis* causes roots to undergo DNA damage that requires homologous recombination and the expression of the cohesin subunit *SYN2* (Sjögren and Larsen, 2017).

The goal of this review is to summarize evidence related to the plant cohesin complex involved in genome maintenance and meiosis. The summary aims to help breeding elite cultivars that will be better adapted to cope with marginal environmental conditions and climate change (Panhwar et al., 2015; Shen et al., 2015), whose crop yields will be high enough to cope with population growth (Gerland et al., 2014), and that may transmit and receive valuable wild alleles efficiently (Dempewolf et al., 2017). The overall aim is to guarantee food security. Of particular interest in modern agriculture is the control of crossover (CO) formation during meiosis, because the low frequency and uneven patterning of COs has traditionally required breeders to work with large populations over many generations to produce desirable haplotypes (Taagen et al., 2020). Fortunately, genome sequencing and advanced genome editing by CRISPR-Cas9 in rice (Li et al., 2020) and maize (Zhang et al., 2020) may enable the translation of knowledge obtained in humans, yeast and *Arabidopsis thaliana*.

## Assembly of Eukaryotic Cohesion and Structural Maintenance of Chromosomes Complexes

Cohesin is an evolutionarily conserved protein complex, believed to assemble as a tripartite ring, that wraps around DNA and modulates its topology (Muñoz et al., 2019). It has a broad range of functions that affect the organization of the eukaryotic genome. The functions include somatic repair of DNA DSBs, regulation of pericentromeric recombination during meiosis I, regulation of transcription, and establishment of sister chromatid cohesion (Fernandes et al., 2019; Muñoz et al., 2019). Biochemical and genetic evidence with baker’s yeast suggest that the canonical cohesin complex contains two structural maintenance of chromosomes (SMC) proteins, SMC1 and SMC3; one α-kleisin subunit named SCC1/RAD21 (mitotic) or REC8/SYN1 (meiotic; Beckouët et al., 2016; Hsieh et al., 2020); and two additional HEAT repeat-containing subunits named SCC3 and PRECOCIOUS DISSOCIATON OF SISTERS 5 (PDS5; Liu et al., 2020). The two SMC proteins assemble as antiparallel coiled coils 50 nm long, with two key features: (1) a hinge domain at one end (N-terminal) and (2) an ABC-like ATPase domain (C-terminal) at the head (Beckouët et al., 2016).

Cohesin stably prevents the sister chromatids from DNA replication until anaphase (G2 to M phases; Elbatsh et al., 2016). In human cells, the loader complex MAU2-NIPBL (SCC2/SCC4 in yeast) loads DNA into the cohesin complex, whereas the activity of the antagonist WINGS APART LIKE (WAPL) releases cohesin from DNA (Faramarz et al., 2020) by opening an exit gate located between SMC3 and the N-terminus of the SCC1 α-kleisin (Elbatsh et al., 2016; Haarhuis et al., 2017). Loading of cohesin by the SCC2/SCC4 loader complex involves stimulation of ATP hydrolysis by the SMC1/SMC3 ATPase domain, which is required for the stable association of cohesin with chromatin (Elbatsh et al., 2016).

**Figure 1:**
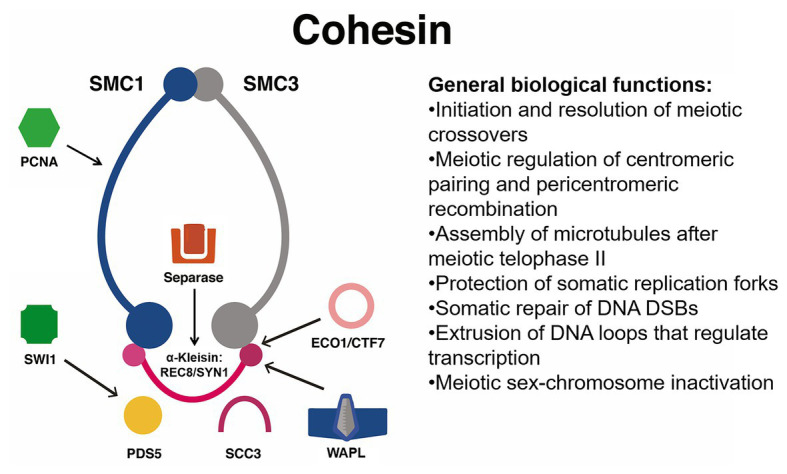
Simplified interaction network between the eukaryotic structural maintenance of chromosomes (SMC) cohesin complex and its functional partners. The plant cohesin complex is proposed to be a ring that stably entraps DNA molecules and facilitates meiotic recombination, centromeric pairing, protection of replication forks, extrusion of chromatin loops, and inactivation of sex chromosomes. These processes are facilitated by locking and unlocking of the ring *via* the simultaneous activity of promoters of cohesion ECO1/CTF7, PDS5, and SWITCH1 (SWI1). Other particularly important factors are PCNA, which facilitates DNA repair and operates as a docking platform for ECO1/CTF7, and cohesin dissociating factors that facilitate dislodging or degradation of the kleisin such as Separase and SCC3 or competitors of PDS5 such as WAPL. Not shown: CTF18. ECO1/CTF7, ESTABLISHMENT OF COHESION 1/CHROMOSOME TRANSMISSSION FIDELITY 7, a N-terminal acetyltransferase; DSB, double-strand break; PCNA, PROLIFERATING CELL NUCLEAR ANTIGEN; PDS5, PRECOCIOUS DISSOCIATION OF SISTERS, a HEAT-repeat protein; REC8, RECOMBINATION PROTEIN 8, a meiotic α-kleisin also known as SYNAPTIC 1 and often abbreviated as REC8/SYN1. The yeast mitotic α-kleisin is known as SCC1, SISTER CHROMATID COHESIN 1, which in the Arabidopsis genome is represented by two *bona fide* orthologs known as SYN2 and SYN4; although a fourth α-kleisin unique to plants called SYN3 is important for both meiosis and mitosis. WAPL, WINGS APART LIKE, a cohesin dissociation factor.

Cohesin may also dissociate from chromatin at anaphase by cleavage of the SCC1 α-kleisin by the separase (ESP), whereas at other stages of the cell cycle, the release of cohesin is mediated by WAPL and the HEAT-repeat subunit PDS5 (Dauban et al., 2020). Repression of cohesion release is enforced during replication by acetylation of two conserved lysine residues within the ATP-ase domain of SMC3, an action performed by ECO1 and assisted by PDS5 (see Table 1; Yang et al., 2019; Dauban et al., 2020). This process stabilizes the entrapment of DNA by cohesin and establishes sister chromatid cohesion throughout the G2 to M phases until cleavage of SCC1 by the separase and deacetylation of SMC3 by HOS1 (Dauban et al., 2020; see Figure 1).

**Table 1:** Selection of cohesin/SMC genes and cofactors with potential for inclusion in breeding initiatives in major crops.

SMC gene and associated factors	Functional features in the literature and public databases^1^^,^^2^	Locus ID as determined by TAIR, NCBI, and the Human Genome Organization (HUGO)^5^	Presumed homologs of interest (rice and maize) retrieved from databases^3^^,^^4^^,^^6^ by the respective coding sequences (CDS)	Likely agricultural use
*Arabidopsis* HORMA-domain protein *ASYNAPTIC 1* (*ASY1*)	Distribution of COs along meiotic chromosomes in a REC8/SYN1 dependent manner	*At1g67370*	*Os09g32930*, *GRMZM2G035996*	Knockdown alleles may reduce linkage drag in major crops
Human cohesin interacting factors *BRG1*/*SMARCA4* and *ARID2* (SWI/SNF subunits)	Cohesin-mediated repression of transcriptional activity, genome maintenance	*HGNC:6597* and *HGNC:196528*	*Os05g05230*, *GRMZM2G102625*, *Os07g33860*, *GRMZM2G009412*	Promotion of seed longevity, intentional induction of inversions and translocations
Human ATP-dependent DNA helicase *DEAD/H-box helicase 11* (*DDX11*)	Synthetically lethal with human *ECO1* homolog *ESCO2* (*HGNC:27230*) and epistatic to human *WAPL* (*HGNC:23293*). Promotes cohesion in arms and centromeres during replication, probably by promotion of SMC3 acetylation, and enhanced stability of replication forks	*HGNC:1663*	*Os05g13300*, *GRMZM2G100067*	Promotion of replication fork stability. May constitute an important layer of genome maintenance under stress across all eukaryotes
*Arabidopsis* N-acetylase *ECO1*/*CTF7*	Acetylation of the ATP-ase domain of SMC proteins, establishment of cohesion, homology-dependent DNA repair. Its function is antagonized by *WAPL*	*At4g31400*	*Os05g31230*, *GRMZM2G100067*; and *Os04g42120*, *GRMZM2G075145*	Prevention of large-scale rearrangements, targeted manipulation of chromosome folding during MSUC and MSCI in meiocytes of papaya, gene expression.
Yeast chromatin-remodeling ATPase *ISW1*/*YBR245C*	Cohesin-dependent processes such as DNA repair and transcription in promoters	*SGD:S000000449*	*Os03g22900*, *GRMZM2G469162*	May stabilize transcription under stress across eukaryotic organisms
Cohesin subunit *PRECOCIOUS DISSOCIATION OF SISTERS 5* (*PDS5A/E*)	Cohesion-promoting cofactor; has a role in fork protection and stable DNA replication	*At5g47690*, *At1g77600*, *At4g31880*, *At1g80810*, and *At1g15940*	For *PDS5A*: *Os06g17840*, *GRMZM2G010637*	May stabilize DNA replication under stress, may be a target for the manipulation of DNA looping and chromosome folding during MSUC and MSCI in meiocytes of papaya
*Arabidopsis SCC2*	Initial CO formation during meiosis, 3D-chromosome folding, loading of cohesin complexes, chromosome looping and DNA repair	*At5g15540*	*Os07g01940*, *GRMZM2G132504*, *Carica papaya evm.model.supercontig_2744.1*	Promotion of crossover resolution, targeted manipulation of chromosome folding during MSUC and MSCI in meiocytes of papaya
*Arabidopsis SCC3*	Regulates release of α-kleisins. Regulates meiotic orientation of kinetochores in *Arabidopsis*, and may have a role in the prevention of large-scale rearrangements during replication, dimerization of cohesin, and chromosome looping	*At2g47980*	*Os05g09620*, *GRMZM2G131443*	May prevent chromosomal rearrangements during replication, in plants, it may provide a valuable layer of genome maintenance under stress
Maize *SCC4/DEK15*	Mitotic chromosome segregation, endosperm and embryo formation, transcriptional regulation of kernel development in maize	*GRMZM2G079796*	*Os04g28010*	Promotion of embryo and endosperm development
*Arabidopsis* condensin subunit *STRUCTURAL MAINTENANCE OF CHROMOSOMES 4* (*SMC4*)	Silencing of pericentromeric TEs, DNA methylation, transcription of genes related to translesion synthesis, male gamete development, tolerance to Boron toxicity	*At5g48600*	*Os05g41750*, *GRMZM2G416501*	May lead to enhanced tolerance to exogenous DNA damage, and improved genome maintenance in crops
*Arabidopsis EXTRA SPINDLE POLES* (*AESP1*), Separase	Cleavage of the REC8/SYN1 α-kleisin during anaphase I and anaphase II, allowing disjunction of homologs and sister centromeres, respectively. Proper assembly of radial microtubule system after telophase II, embryo development, cellularization of the endosperm, and vesicle trafficking	*At4g22970*	*Os02g53120*, *GRMZM2G300624*	Promotion of crossovers resolution and proper tetrad formation. May facilitate breeding of fertile cultivars in major crops
*Arabidopsis SWITCH1/DYAD* (*SWI1*)	Sister chromatid cohesion and meiotic chromosome organization, assembly of the chromosome axis	*At5g51330*	Known as *AMEIOTIC* (*OsAM1*) in rice: *Os03g44760*, and *AMEIOTIC* (*AM1*) in maize (possibly *GRMZM5G883855*)	May facilitate the identification of new alternative meiotic α-kleisins in crops
*Arabidopsis WINGS APART-LIKE PROTEIN 1* (*WAPL1*/*2*)	Release of meiotic α-kleisin REC8/SYN1 during prophase I. Cohesin removal by WAPL is required to complete DNA synthesis under conditions of persistent DNA replication stress. Regulates chromosome folding	*At1g11060* and *At1g61030*	*Os10g35380, GRMZM2G034276*; *Os10g35380*, *GRMZM2G034276*	New alleles may facilitate DNA replication under persistent damage. May confer tolerance to chronic DNA damage in crops

MSUC, meiotic silencing of unsynapsed chromatin; MSCI, meiotic sex chromosome inactivation; TE, transposable elements.

1 Database consulted was The Arabidopsis Information Resource (TAIR): https://www.arabidopsis.org/.

2 Database consulted was National Center for Biotechnology Information (NCBI): https://www.ncbi.nlm.nih.gov/.

3 Database consulted was Phytozome v12.1: https://phytozome.jgi.doe.gov/pz/portal.html.

4 Database consulted was Maize GDB: https://www.maizegdb.org/.

5 Database consulted was the Human Genome Organization (HUGO): http://www.hugo-international.org/.

6 Database consulted was the Rice Genome Annotation Project: http://rice.plantbiology.msu.edu/cgi-bin/gbrowse/rice/.

According to the multi-state asymmetric ATPase cycle, the three known SMC complexes – cohesin SMC1/SMC3, condensin SMC2/SMC4, and SMC5/SMC6 complexes – have a ground state known as the nucleotide-free state, in which only one SMC head can bind to an ATP molecule. Upon binding of the first ATP molecule to the P-loop of the RecA lobe, the glutamine residue in the Q-loop changes its position and establishes hydrogen bonds with γ-phosphate and with an Mg^2+^ ion. As a result, the helical lobe tilts by 15°, repositions a tyrosine-lysine pair within the W-loop and causes dissociation of PDS5 from SMC1 (its SMC4 counterpart is YCS4; Hassler et al., 2019). Binding of a second ATP molecule causes the formation of a quasi-symmetric SMC1/SMC3 (or SMC2/SMC4) head dimer with both RecA active sites occupied by ATP molecules and induces the coiled-coils of SMC1 (or SMC2) to spread apart and dissociate from the SCC1 kleisin (BRN1 in the yeast condensin complex). This model implies that the structural transitions in SMC complexes are governed by nucleotide release, as observed in other ABC-type ATPases. It hints at the existence of directionality in the translocation of the SMC holocomplexes along the DNA double helix (Hassler et al., 2019). In plants, these results should be validated to develop a better understanding of the torsion of the W-loop of SMC1 and how it controls the dissociation of cofactors such as PDS5 during plant meiosis, for instance. Such validation might also open avenues for engineering changes in the rate of ATP-hydrolysis to cope with faster structural transitions under chronic DNA damage stress (see Table 1).

## Engineering of Cohesion, Supercoiling, and Cell Cycle Progression

During DNA replication in yeast, instead of entrapping just one DNA molecule, cohesin holds together two sister DNAs (Liu et al., 2020). Experimental evidence in yeast strain W303 suggests that this might be achieved in two ways: (1) the replisome is able to replicate through cohesin rings or (2) cohesin transiently loses contact with DNA and is reloaded behind the replication fork. In addition to the essential ECO1 acetyltransferase, several other non-essential replisome components contribute to the establishment of sister chromatid cohesion, such as the downstream Ctf18-replication factor-C (Ctf18-RFC) complex. Ctf18-RFC is a member of the RFC group, which are complexes of pentameric AAA^+^ ATPases that load and unload PCNA sliding clamps and function as DNA-replication checkpoint factors. PCNA is involved in a wide array of DNA functions, mostly as a processivity factor for DNA polymerases but also as a docking platform for other proteins, possibly including ECO1. Yeast cells lacking the Ctf18 subunit show impaired SMC3 acetylation (K112 and K113) as well as cohesion defects. Notably while these defects are reversed by deletion of the PCNA unloader ELG1-RFC, the DNA replication checkpoint response only worsens (Liu et al., 2020). In *Arabidopsis*, *CTF18* may have a role in the establishment of cohesion and plant growth but not in somatic DNA repair (Takahashi et al., 2010). New alleles may need to be developed by CRISPR-Cas9 to fully characterize the function of *CTF18* in plants. The maize *SMC3* homolog has been successfully characterized with genome editing by CRISPR-Cas9 (Zhang et al., 2020). The homolog fulfills a crucial role during the establishment of centromeric pairing during meiosis and sister chromatid cohesion during mitosis (in root tips; Zhang et al., 2020). So, maize might be amenable to the study of SMC3 acetylation dynamics during meiosis and mitosis and perhaps lead to the discovery of novel phenotypes.

In yeast, a mechanism that links DNA replication and SMC3 acetylation has been characterized (Liu et al., 2020). CTF18 works upstream of SMC3 acetylation by facilitating loading of PCNA onto the leading strand, where it can face ECO1/CTF7 during the late stages of DNA replication, possibly by stimulating interaction at the PCNA interacting peptide (PIP) box of ECO1/CTF7 (QxxLxxFF; Moldovan et al., 2006; Liu et al., 2020). As a result, ECO1/CTF7 becomes well-placed behind the replication fork, where it can better acetylate freshly deposited cohesin, which causes both sister DNA molecules to become embraced by cohesin (see Table 1; Liu et al., 2020; see Figure 1). The cohesion defects present in yeast *ctf18* cells can be overcome by the *wapl* allele (Liu et al., 2020; Table 1), a result also observed in *Arabidopsis ctf7/wapl* mutants (De et al., 2016). Hence, in plants, weak *wapl* alleles may facilitate cohesion under replicative stress, although fertility may be compromised (De et al., 2016). Fusion of the PIP box to the PCNA also restores growth in the presence of ECO1^-pip^ (Liu et al., 2020). Comparison of DNA repair efficiencies and plant growth in *wapl* mutants and plants engineered to carry new PCNA-ECO1 modules may be an interesting research topic in plants exposed to chronic DNA damage, including crops exposed to high doses of UVB radiation such as high-altitude maize (Rius et al., 2016) or plants grown in the aluminum-rich, acidic soils of most developing countries (Chen et al., 2019b).

## Pathways To Achieve Robust Transcription During Plant Mitosis and Meiosis: New Roles For Cohesins and SMC5/SMC6 Complexes

In addition to its role in establishing sister chromatid cohesion, the cohesin complex is also believed to mediate transcription, and it is recruited to sites of DNA DSBs to promote repair during the S and G2 phases of the cell cycle (Dorsett and Ström, 2012; Meisenberg et al., 2019). In response to a DNA DSB, eukaryotic cells usually respond by repressing transcriptional activity in neighboring chromatin. This pathway depends on the ATAXIA-TELANGIECTASIA MUTATED (ATM) kinase and involves the activity of chromatin remodeling complexes of the SWItch/sucrose non-fermentable (SWI/SNF) family that ubiquitylate H2AK119 at sites of damage. Recently, cohesin was found required for the activity of SWI/SNF complexes after DNA damage, mainly at centromeres. Repression of transcription by subunits BRG1 and ARID2 of the mammalian PBAF complex (a SWI/SNF complex; see Table 1) contributed to genome stability by preventing the formation of large-scale rearrangements during the S, G1, and G2 phases in the prostate cancer cell line LNCaP (Meisenberg et al., 2019). This novel function may not depend on the presence of a sister chromatid and may operate differently from non-homologous end joining or homologous repair. Cohesin may loop DNA in areas flanking the break to reorganize the chromosome. This situation may prevent transcription and remove the broken DNA end from the vicinity of other actively transcribed genes to prevent misrepair (Meisenberg et al., 2019). Notably, subunit SA2 (SCC3) and ECO1 are crucial for this process (see Table 1), but WAPL is dispensable (Meisenberg et al., 2019), which suggests that in human cells, SA2/SCC3 is a true tumor suppressor gene. In yeast, the activity of SCC3 is required for the release of the mitotic α-kleisin SCC1 (known as SYN2 and SYN4 in *Arabidopsis*), while during meiosis in *Arabidopsis*, the activity of *Arabidopsis* SCC3 is essential for the proper orientation of kinetochores (Chelysheva et al., 2005; Yuan et al., 2014; Beckouët et al., 2016). Further research could investigate whether SCC3 might also have a role in preventing rearrangements during the mitotic and meiotic S-phase.

To our knowledge, this SWI/SNF and SCC3-mediated mechanism of preventing large-scale re-arrangements has not been characterized in *A. thaliana* or in crops such as rice or maize and might constitute an entirely new system of DNA repair that may lead to far-reaching discoveries in plant developmental biology and plant breeding. For instance, genome maintenance is crucial for seed longevity (Waterworth et al., 2019), so this topic seems promising in plant breeding and plant science because it may allow for the engineering of ultra-long seed storage.

**Figure 2:**
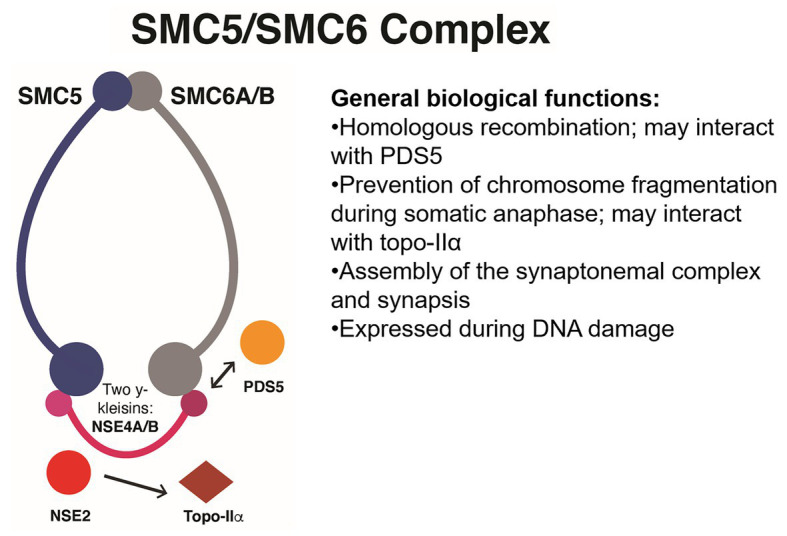
Simplified functional interaction network of the SMC5/SMC6 complex in genome maintenance, homologous recombination, and meiosis. These SMC complexes interact with PDS5 during homologous recombination and have an especially important role during meiotic synapsis as well as somatic chromosome segregation. These complexes may also be required for proper somatic DNA repair *via* the SUMOylation of topoisomerase IIα by associated protein NSE2. NSE proteins have a wide variety of functions that affect pollen development and homologous DNA repair. Topo-IIα, topoisomerase-IIα.

Coincidentally, the activity of the SMC5/SMC6 complex is crucial for successful maintenance of the genome during interphase in cancer (HCT116) and immortalized non-cancer cells (RPE1; Venegas et al., 2020). DNA damage is increased in cells deficient in SMC5/SMC6 function that are stained with the DNA damage marker γ-H2AX, which suggests that SMC5/SMC6 is required for proper homologous recombination. Also during the S-phase, activity of the SMC5/SMC6 complex is required to prevent fragmentation of chromosomes later on during anaphase (Venegas et al., 2020). This second requirement might be linked to the role of the SMC5/SMC6 subunit NON-SMC ELEMENT2 (NSE2) during SUMOylation of topoisomerase IIα (Venegas et al., 2020). In general, the activity of the SMC5/SMC6 complex could be a target for plant breeding, specifically for selecting seed longevity and smooth endosperm proliferation. The *Arabidopsis* genome contains one SMC5 and two SMC6 homologs (SMC6A/B) as well as two γ-kleisins, NON-SMC ELEMENT4 A/B (NSE4A/B), and five other NSE subunits (see Figure 2). Their combined activity is required for proper seed development and for efficient homologous recombination in somatic tissues (Watanabe et al., 2009; Díaz et al., 2019; Zelkowski et al., 2019). Functional characterization of NSE4A/B indicates that their activity is required for pollen development, tolerance to the radiomimetic agent Bleomycin, proper chromosome segregation during meiosis, and proper assembly of the synaptonemal complex (Hesse et al., 2019; Zelkowski et al., 2019). The association between the SMC5/SMC6 complex, topoisomerase IIα, and prevention of mitotic chromosome fragmentation has not been functionally characterized in crops.

## The Role of SMC Complexes in DNA Repair: Opportunities For Engineering Tolerance To DNA Damage

In humans, chronic DNA replication stress is a source of generalized DNA damage and a hallmark of cancer (Macheret and Halazonetis, 2015; Minchell et al., 2020), but in plants, little is known about the impact of chronic DNA replication stress or chronic DNA damage. Work in *Arabidopsis* suggests that a low dose of gamma radiation (2000 μGy h^−1^ for 54 days) is enough to reduce plant growth by 50% (Vandenhove et al., 2010). Exposure to a high dose (200 Gy) at the early reproduction stage (33 days after sowing) led to a robust increase in the expression of genes for homologous DNA repair, such as *PROLIFERATING CELL NUCLEAR ANTIGEN 1/2* (*PCNA1/2*, At1g07370, and At2g29570) and *POLY(ADP-RIBOSE) POLYMERASE 2* (*PARP2*, At4g02390; Kim et al., 2007). In *P. sativum*, seedlings irradiated with 0.4–10 Gy showed reduced pod set by 20–40%, but with >10 Gy, they were no longer able to flower. At all doses, male meiotic tetrad formation was defective, as evidenced by the formation of micronuclei at a rate of 10%. All F_2_ plants from irradiated F_1_ individuals showed reduced pod set and a significant formation of meiotic micronuclei (Zaka et al., 2004). Evidence in yeast suggests that DNA damage related to DNA replication is prevalent within genomic contexts that are not conducive to normal progression of the replication fork. Structures that block free rotation of DNA also cause accumulation of topological stress and exceed the relaxation activity of topoisomerases. Impaired progression of the replication fork caused by topological stress may lead to fork reversal, to stabilize an arrested fork, or to fork rotation that transforms overwinding ahead of the fork into precatenates behind the replication fork and thus allow elongation without intervention by topoisomerases (Minchell et al., 2020). Thus, further study is needed to understand how to facilitate replication while reducing topological stress and damage, possibly as a strategy to assist plant growth at critical moments such as during cotyledon emergence and seed ripening.

Work in budding yeast indicates that centromeres, telomeres, long terminal repeat sites, replication origins, and rDNA repeats consistently accumulate DNA damage, as seen by enrichment of γ-H2AX on ChIP-chip experiments (Szilard et al., 2010). In budding yeast, the cohesin complex promotes genome stability following replication stress, especially during the S phase when it is preferentially loaded onto centromeres and rDNA sites (Frattini et al., 2017; Minchell et al., 2020). However, paradoxically, the cohesin complex could also represent a large barrier to the diffusion of topological stress by preventing free rotation of DNA (Minchell et al., 2020). In fact, depletion of SCC1 in topoisomerase IIα-depleted yeast cells (strain W303-1a) suppresses the accumulation of γ-H2AX across centromeres and rDNA sites, whereas depletion of ECO1/CTF7 partially reduces γ-H2AX accumulation in centromeres. Surprisingly, depletion of the condensin SMC2 subunit reduces the abundance of the γ-H2AX mark in rDNA, which suggests that both SMC holocomplexes affect the accumulation of DNA damage following topological stress, perhaps as a mechanism to prevent ectopic recombination in rDNA sites and maintains the bi-orientation of centromeres stress (Minchell et al., 2020). In fact, use of the yeast mini-chromosomes YCp50 and YRp21 has shown that inactivation of *SMC2* leads to increased formation of DNA knots generated by the activity of topoisomerase IIα, possibly by affecting condensin-mediated loop extrusion (Dyson et al., 2021; see Table 1). This type of work has not been validated in plants, but double or triple mutants might be obtained in *Arabidopsis*. If so, the impact of increased topological stress in centromeres during replication could be determined by characterizing alleles that might make the process more robust during plant meiosis and mitosis.

**Figure 3:**
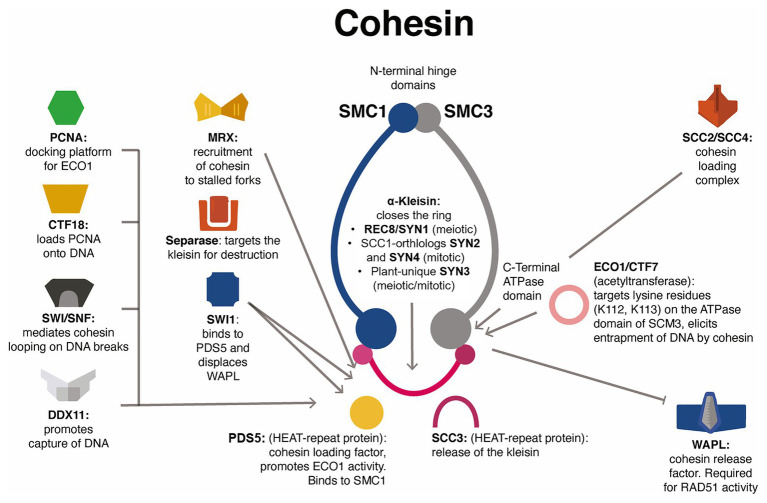
Proposed simplified functional network between the plant cohesin complex and associated genome-maintenance factors. The cohesin complex performs a wide repertoire of functions including homologous recombination during meiosis and mitosis. The complex is loaded onto chromatin by SCC2/SCC4; it is stably locked onto chromatids by ECO1/CTF7, an acetyltransferase that targets lysine residues on the ATPase domains of SMC3 (and possibly SMC1); and entrapment of DNA is promoted by factors such as PDS5 and SWI1 and antagonized by WAPL and SCC3, which may target the meiotic α-kleisin REC8/SYN1 or the mitotic α-kleisin SCC1, which in *Arabidopsis* is represented by orthologs SYN2 and SYN4, while SYN3 is unique to plants and is both meiotic and miotic. The Separase (ESP1 in *Arabidopsis*) also targets kleisins for degradation. Several other factors promote its function, such as PCNA during homologous recombination; CTF18, which might promote its loading onto DNA; DDX11, which might promote capture of DNA; SWItch/sucrose non-fermentable (SWI/SNF) complexes that promote cohesin-mediated looping on DNA breaks; and the MRX complex that promotes recruitment of cohesin on replication forks that stall. Characterization of these processes in plants may facilitate the understanding of meiosis, DNA repair, and genome organization.

In human cells, the super family 2 (SF2) helicase DEAD/H-box helicase 11 (DDX11, named Chl1 in budding yeast) is synthetically lethal with ESCO2, and knockdown of WAPL partially restored cohesion defects in DDX11-deficient cells (see Table 1). In humans, loss of DDX11 activity causes the cohesion syndrome Warsaw Breakage syndrome, whereas loss of ESCO2 causes Roberts syndrome. DDX11 interacts with several replication factors, such as PCNA, the 5'-flap endonuclease Flap Structure-Specific Endonuclease 1 (FEN1), the fork protection complex subunit Timeless, and CTF4, which links the activity of the Minichromosome Maintenance Protein Complex (MCM) helicase with DNA polymerases (Faramarz et al., 2020). DDX11 is believed to specialize in resolving DNA structures such as forked duplexes, 5′-flap duplexes, and anti-parallel G-quadruplexes, therefore, promoting replication fork progression (Faramarz et al., 2020). DDX11 may promote the establishment of cohesion in three ways: (1) subtle promotion of SMC3 acetylation, (2) promotion of cohesin activity by facilitating second-strand capture, and (3) enhanced overall replication fork stability (see Figure 3). Rescue of cohesion in *DDX1*-deficient cells by knockdown of *WAPL* suggests that the helicase may facilitate chromatid cohesion in arms and centromeres (Faramarz et al., 2020). Once again, promotion of cohesion occurs in *ddx1* and *wapl* mutants could be tested in *Arabidopsis* or in crops such as rice and maize, as well as whether this genetic interaction may protect against chronic DNA damage (see Table 1).

In budding yeast, a strong link has also been observed between the establishment of cohesion, replicative stress during the S-phase, and nuclease activity by the Mre11-Rad50-Xrs2/Nbs1 (MRX/N) complex. Several other factors may participate as well, such as the histone remodelers Chd1 and Isw1. Collapse of replication forks and replication resumption requires extensive processing of stalled forks by DNA helicases, endo- and exonucleases, and recombinases. Of great importance for this process is the activity of the MRX/N complex, which is known for its role in repair of DSBs. At DNA DSBs, the endo- and exonuclease catalytic activity of MRE11 generates gaps seen as short single-strand DNAs that are then extended by the Exo1 nuclease or the Sgs1 complex. Under replication stress conditions, the MRX/N complex facilitates repair *via* recombination of damaged forks by resecting available nascent DNA strands. In budding yeast, the MRX/N complex promotes recruitment of cohesin to stalled forks to facilitate sister-chromatid recombination required for fork restart (Delamarre et al., 2020; see Figure 3). The exact mechanism by which the complex promotes the loading of cohesin to stalled forks is currently unknown (Delamarre et al., 2020), and their precise role in establishing cohesion in plants is ignored. In *Arabidopsis*, *mre11* mutants show chromosomal fragmentation during mitosis and meiosis and do not exhibit synapsis during meiosis. Plants are small, misshapen, and sterile (Puizina et al., 2004). Facilitating fork restart under active transcription might prove valuable to guarantee and boost growth in quickly developing tissues and organs such as flowers, fruits, and grains. Novel gain-of-function alleles may need to be developed in *Arabidopsis* and crops.

In yeast, the DSB processing activity of the MRX complex leads to the formation of excess single-strand DNA coated with replication protein A (RPA), which triggers the recruitment of the Mec1 kinase (ATAXIA-TELANGIECTASIA AND RAD3-RELATED, ATR in *Arabidopsis*; Bourbousse et al., 2018; Delamarre et al., 2020). In *Arabidopsis*, analysis of γ-H2AX foci in *mre11* and *rad50* mutants suggested that DNA damage signaling in these plants is ATR-dependent, may facilitate recombination at stalled or collapsed replication forks, and may affect processes such as telomere maintenance, cell cycle regulation, and cell proliferation in roots (Amiard et al., 2010). In fact, in *Arabidopsis*, the ATM and ATR kinases are involved in targeting the master regulator of the DNA damage response, the SUPPRESSOR OF GAMMA RESPONSE 1 (SOG1) transcription factor, which then controls the expression of SMC5/SMC6, SYN2, SCC2, ASY3, and NSE4A (Yoshiyama et al., 2014; Bourbousse et al., 2018). This pathway regulates responses to Al^3+^ toxicity and DNA damage in roots of *Arabidopsis*. RAD51-dependent, homology-mediated repair is involved in these responses to Al^3+^ (Chen et al., 2019b). Further study could determine whether MRX/N complexes in conjunction with cohesin and other SMC complexes control tolerance to DNA damage by Al^3+^ in crops such as rice.

In humans, removal of cohesin by WAPL-and PDS5-dependent cohesin is important for fork protection and smooth DNA replication (Benedict et al., 2020). Tightly regulated cohesin loading and unloading occurs under conditions of DNA replication stress, characterized by stalling replication forks that frequently collapse and cause DNA DSBs (Benedict et al., 2020). In human cancer cells, a decrease in sister chromatid cohesion is a common trait resulting from oncogenic pathway activation and DNA replication stress (Benedict et al., 2020). Moreover, in the absence of *WAPL* or *PDS5B*, mouse embryonic fibroblasts with inactivation of *Retinoblastoma* (*Rb*; TKO-Bcl2-p53KO) and deprived of mitogens fail to repair DSBs at collapsed replication forks, which leads to reduced proliferation and increased apoptosis (see Table 1; Benedict et al., 2020). Increased cohesin removal by WAPL in human HAP1 cells is required to complete DNA synthesis under conditions of persistent DNA replication stress, probably by allowing RAD51-dependent repair of forks. This condition may also cause premature loss of sister chromatid cohesion during mitosis (Benedict et al., 2020). Whether overexpression of *WAPL* in *Arabidopsis* or crops may confer enhanced tolerance to DNA damage by UVB or Al^3+^ is unclear.

## Cohesins, Chromosome Folding, and Meiotic Silencing of Unsynapsed Chromatin: Engineering Sex Determination in Crops

**Figure 4:**
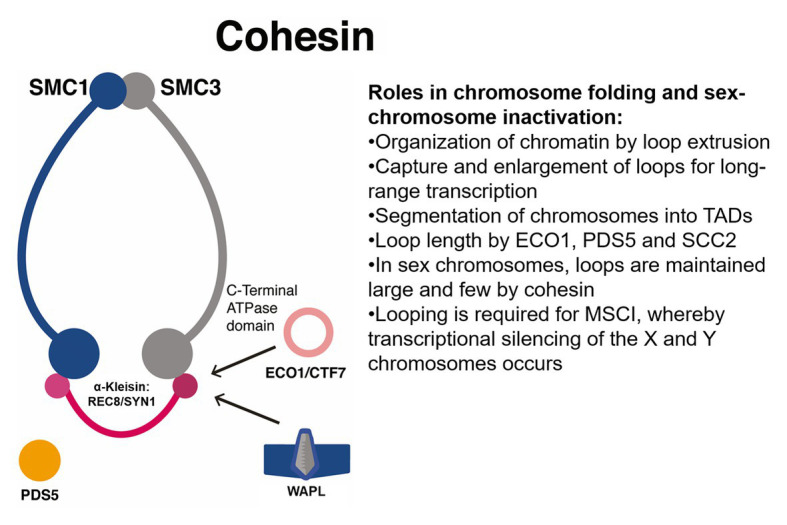
Proposed roles for the SMC cohesin complexes in meiotic silencing of unsynapsed chromatin in sex chromosomes. Cohesin has roles in the organization of chromatin into loops that regulate expression and in sex chromosomes, may enforce silencing. ECO1/CTF7, N-terminal acetyltransferase ESTABLISHMENT OF COHESION 1/CHROMOSOME TRANSMISSSION FIDELITY 7; MSCI, meiotic sex chromosome inactivation; PDS5, HEAT-repeat protein PRECOCIOUS DISSOCIATION OF SISTERS; TADs, topologically associating domains; WAPL, cohesin dissociation factor WINGS APART LIKE.

In yeast, the interplay between cohesin, ECO1, PDS5, and WAPL is complex, and the processes regulated by this interplay may also extend into chromosome folding, a process that is associated with condensin-dependent formation of loops that then facilitate folding of mitotic chromosomes into compact structures (Dauban et al., 2020). However, cohesin rings organize DNA within chromatids *via* a loop extrusion process, in which small loops are captured and enlarged into large Mb-sized structures that contribute to long-range gene regulation during interphase, not compaction. This process also segments interphase chromosomes into topologically associating domains (TADs; Dauban et al., 2020). TADs also exist in *Nipponbare* rice and cover about one quarter of the genome. The boundaries feature (1) CG and CHG methylation; (2) euchromatic histone marks H3K4me2/3, H3K9ac, H3K36me3, and H3K12ac; and (3) active expression; and (4) are enriched with a GC-rich motif that is recognized by transcription factors of the TEOSINTE BRANCHED 1, CYCLOIDEA, PCF1 family (Liu et al., 2017a). The length of cohesin-mediated loops is considered to depend on the residence time of cohesin in DNA and a poorly understood extrusion driving force, perhaps related to SCC2-mediated ATP-hydrolysis (see Table 1), pushing of cohesin along the DNA by RNA polymerases or the frequency of cohesin injection. Hi-C maps of mitotic chromosomes from yeast cells (strain W303) depleted in CDC45 (which reach mitosis without replication) suggest that the establishment of loops is independent of sister chromatid cohesion. Inactivation of WAPL and PDS5 activity in mammalian cells abolish cohesin turnover and lead to an increase in loop length (see Figure 4). In yeast, the activity of ECO1 inhibits the loop translocation process that extends DNA loops. Moreover, depletion of ECO1 in yeast *wpl1Δ* cells promotes long intra-chromosomal contacts identical to those observed in PDS5-depleted cells. Hence, in yeast, PDS5 may recruit both ECO1 and WAPL to cohesin and the length of loops may be regulated by two independent pathways controlled by ECO1 and WAPL (see Table 1; see Figure 4; Dauban et al., 2020).

In mammals, deposition of chromosome axis proteins is required for meiotic silencing of unsynapsed chromatin (MSUC), the process whereby unsynapsed chromosomal regions in the Y chromosome undergo transcriptional inactivation during prophase I. This process constitutes a meiotic checkpoint activated in response to the presence of partially or completely unsynapsed regions such as extra chromosomes or chromosomal translocations and triggers epigenetic silencing. A related process is meiotic sex chromosome inactivation (MSCI), whereby transcriptional silencing of the X and Y chromosomes occurs during prophase I (see Figure 4). Usually, DSBs generated in leptotene are repaired during zygotene *via* homology search, but for sex chromosomes, large portions do not synapse, and thus DSB markers (such as RPA and RAD51) accumulate along their axes. The presence of such markers induces the ATM/ATR pathway to trigger two rounds of histone H2AX phosphorylation (γ-H2AX) deposition that coats both the X and Y chromosomes. The presence of γ-H2AX is followed by the accumulation of repressive histone marks such as H3K9me3/2, H2A ubiquitylation, H3K27m1/3, H3K9ac, and H4K16ac and the absence of active RNA polymerase II (Waters and Ruiz-Herrera, 2020). By Hi-C analysis in mice germ cells, MSCI was linked to 3D higher-order chromatin remodeling, which features reduced loading of meiotic cohesin α-kleisins REC8 and RAD21 Cohesin Complex Component Like 1 (RAD21L) and the organization of sexual chromosomes into TADs (see Figure 4). Sex chromosomes feature few and large loops and significant clustering of cohesins into the promoter regions of highly expressed genes most probably located in DNA loops out of the axes (Vara et al., 2019; see Figure 4).

In mice, MSCI is essential for avoiding ectopic expression of male “pachytene-lethal” genes (also known as “executioner” genes). These are Y-linked genes that encode zinc finger transcription factors, which, when expressed, induce pachytene arrest (Waters and Ruiz-Herrera, 2020). Notably, the tropical crop and emerging model system *Carica papaya* Linn features a sex chromosome system but with two slightly different Y chromosomes: Y for males and Y^h^ for hermaphrodites (Liao et al., 2017; Lee et al., 2018). Papaya has three sex types: female (XX), male (XY), and hermaphrodite (XY^h^), males being rare because the trait is usually lethal (Chen et al., 2019a). The hermaphrodite-specific region of the Y^h^ chromosome (HSY) is 8.1 Mb long and the X chromosome is 3.5 Mb long (Liao et al., 2017) and heavily methylated (Zhang et al., 2008). Papaya is an important source of vitamin A, vitamin C, potassium, folate, niacin, thiamine, riboflavin, iron, and calcium. Clearly, suppression of recombination and degeneration of the Y chromosome occurs (Zhang et al., 2008; Charlesworth, 2017). Therefore, Hi-C could be used with papaya meiocytes to determine whether MSUC and MSCI occur and whether cohesins and especially PDS5 are important for such processes. Such research may help clarify how sex is determined in papaya, perhaps by differential loading of cohesins in DNA loops or by differential activity of ECO1 and WAPL. This type of work may allow for the deliberate engineering and breeding of constitutively hermaphrodite cultivars, the most agriculturally desirable (Lee et al., 2018; see Table 1). Constitutively hermaphrodite papaya cultivars are known to occur occasionally in farms. One example is the all-hermaphrodite progeny of BH*-TSS No. 7, an inbred line derived from the rare X chromosome mutant *S*, which may contain a recessive lethal allele, ml, on the X chromosome (Chen et al., 2019a).

## The Role of ECO1 Complementation Group in Mitosis and Meiosis: Opportunities For Plant Breeding

In yeast, from the S-phase onward, cohesin stably holds together the sister chromatids. For this, cohesin must be protected from removal by WAPL. Protection is provided by the ECO1 acetyltransferase, which performs this task by targeting the SMC3 subunit at two highly conserved lysines on the outside of its ATPase domain, and as a result, locks cohesin and makes it resistant to WAPL (see Table 1; Elbatsh et al., 2016).

A genetic screen of about 500 *eco-1* mutants in haploid yeast strain W303 allowed for the identification of a complementation group that had no identifiable mutations in *WAPL*, *SMC3*, *SCC3*, and *PDS5* (Elbatsh et al., 2016). The SMC1 mutation L1129V, located in the signature motif, is an integral part of the ATP binding pocket, and D1164E is part of the D-loop, a motif required for the hydrolysis reaction and the proper orientation of another loop, the P loop, which is required for proper nucleotide binding (Elbatsh et al., 2016; Hassler et al., 2019). Although these mutations in SMC1 allow for binding to DNA in the absence of ECO1 and confer cohesion and viability, scanning force microscopy results suggest the mutants are unable to hydrolyze ATP, and the overall amount of cohesin is reduced by 30%. The analogous SMC3 mutation L1126V partially supports viability, whereas the SMC3 mutation D1161E is lethal. The SMC1 mutation L1129V shows impairment in the opening of the cohesin exit gate due to failure to properly orientate the ATPase for hydrolysis (Elbatsh et al., 2016). This mechanism seems to be conserved in human cells (HCT116 *p53*^−/−^) cells (Elbatsh et al., 2016), but has not been studied in plants. In yeast (strain W303), the acetylation of the ATPase heads by ECO1 is believed to occur preferentially during a conformation called the J mode (SMC1-S161/SMC3-K160), which allows for better entrapment of sister DNAs and is characteristic of cohesion (Chapard et al., 2019). Hence, mutations within the D loop of SMC1 (L1129V and D1164E) and in the J compartment need to be isolated in *Arabidopsis* to determine the corresponding phenotypes. Most likely, these might correspond to weak alleles.

In yeast, ECO1 has been proposed to interact physically with PCNA through the PIP box (also known as the QxxL motif). Introducing the alternate sequence AxxA causes cohesion defects (Moldovan et al., 2006). Fusion of the PIP box directly to the PCNA restores cell growth, sister chromatid cohesion, and SMC3 acetylation in yeast strain W303. Therefore, a possible explanation is that ECO1 is placed close to the PCNA during the late stages of DNA replication to function during the establishment of cohesion. A proposed mechanism is that the CTF18-RFC complex may operate upstream of this process by loading the PCNA at DNA replication forks, away from sites of active replication, possibly in a post-replicative manner (Liu et al., 2020). In yeast, the role of the PIP box during the establishment of cohesion has been thoroughly characterized, especially the role of post-translational modifications such as SUMOylation (Moldovan et al., 2006), and performing similar studies in plants might be scientifically relevant.

In yeast (YSD17 and several other haploid strains) and human cells (HeLa, 293 T), self-interaction of the cohesin subunits SCC1/RAD21 and SCC3 causes cohesin to dimerize in the S phase and monomerize in mitosis (see Table 1). Also, deletion of the deacetylase HOS1 (which erases SMC3 acetylation) or the ECO1 antagonist WAPL1 could increase cohesin dimer levels by 20%, whereas depletion of ECO1 had the opposite effect. Dimerization of SMC complexes (cohesin and condensin) is considered as a key for DNA loop formation (Shi et al., 2020), so ECO1/CTF7 appears indispensable for high-order chromosome structure and gene expression (see Table 1).

In *Arabidopsis*, *ECO1/CTF7* regulates tolerance to DNA damage, pairing at zygotene, proper segregation of meiotic and mitotic chromosomes at anaphase, tapetum integrity, mitotic cell cycle progression, somatic DNA repair, root development, pollen mitosis, seed development, and chromatin condensation (Bolaños-Villegas et al., 2013), in a manner that is epistatic to *WAPL1/2* (De et al., 2016). However, we have no biochemical information regarding its interaction with SMC3, SCC3, and PDS5 or the PCNA, and its role in CO formation is ignored as well. Rice has two *ECO1/CTF7* homologs that might be essential for the proper acetylation of SMC3 (K105 and K106) during meiosis. Thus, genome editing by CRISPR-Cas9 may facilitate the retrieval of suitable mutant alleles (Li et al., 2018).

Five homologs of *Arabidopsis PDS5* have been characterized. In the *Atpds5a/Atpds5b/Atpds5c/Atpds5e* mutant, the impact on meiosis is restricted to a minor reduction in chiasmata formation in chromosome 1, but the *Atpds5a/Atpds5b/Atpds5c* mutant shows significantly impaired somatic homologous repair after exposure of seedlings to Bleomycin (5 μg/ml), as observed with the *IC9* recombination reporter, in which the recombinant sectors appear blue after *GUS* staining. Double, triple, and quadruple mutants are also sensitive to γ-radiation (150–450 Gy) and the cross-linking agent mitomycin C. The authors indicated that this phenotype is similar to that reported for *Arabidopsis SMC6*, which may suggest a functional relationship between PDS5 and the SMC5/SMC6 complex (Pradillo et al., 2015).

In conclusion, the activity of *ECO1/CTF7* and its interacting partners PDS5 and WAPL is required for overall plant development, including meiosis, mitotic DNA repair, and most likely gene expression. It may be necessary to isolate mutants in crops to identify novel phenotypes relevant to plant breeding and perhaps determine whether these genes behave as a narrow complementation group as reported in yeast (Elbatsh et al., 2016).

## Engineering Systems Redundancy: Complementary Roles of Cohesin and Condensin in Plant Genome Organization, Silencing of Transposons, and Tolerance To DNA Damage

Eukaryotes have three sets of canonical SMC protein complexes (Municio et al., 2021): (1) the cohesin complex composed of the core proteins SMC1 and SMC3, mentioned previously, mostly involved in sister chromatid cohesion; (2) the SMC5/SCM6 complex, which is mostly linked to DNA repair and recombination; and (3) the condensin complex, which is composed of core units SMC2 and SMC4 and is required for chromosome condensation and segregation (Sakamoto et al., 2019).

**Figure 5:**
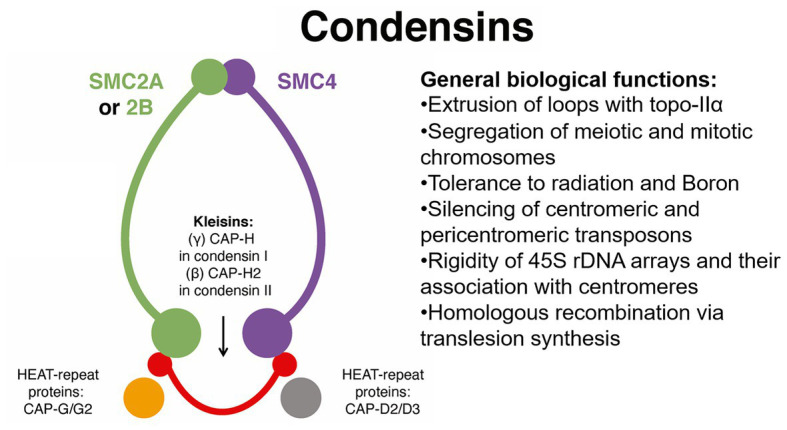
Simplified functional network for SMC condensin complexes during genome maintenance and organization. The plant condensin complexes are versatile and regulate a wide range of processes including chromatin loop extrusion, chromosome segregation, silencing of centromeric transposable elements, organization of 45S ribosomal DNA arrays, and homologous recombination that affect pollen and embryo development. Much of this activity is facilitated by HEAT-repeat proteins CAP-G/G2 and CAP-D2/D3. Topo-IIα, topoisomerase-IIα.

In *A. thaliana*, condensin is further divided into complexes I and II (Sakamoto et al., 2019). The plant condensin complex I consists of core units SMC2/SMC4, γ-kleisin (CAP)-H, β-kleisin (CAP)-H2, and HEAT repeat-containing proteins CAP-G and CAP-D2. However, the association of SMC2/SMC4 with CAP-H2 (β-kleisin), CAP-G2, and CAP-D3 form condensin II (Sakamoto et al., 2019; Municio et al., 2021), a complex that may be required for tolerance to DSBs caused by the radiomimetic agent zeocin for tolerance to boron (Sakamoto et al., 2011; see Figure 5). In *Arabidopsis*, defects in the function of *SMC2* (2 genes) or *SMC4* (3 genes) affect segregation of mitotic and meiotic chromosomes (Sakamoto et al., 2019). Also, defects in *Arabidopsis SMC4* (At5g48600, also known as *AtCAP-C*) cause deregulation in the expression of centromeric and pericentric transposable elements (such as *COPIA*), reduced CG methylation, and chromocenter decondensation (see Table 1), phenotypes reminiscent of those reported for *ECO1/CTF7* mutants (Bolaños-Villegas and Jauh, 2015; De et al., 2016) and perhaps suggesting a regulatory link between SMC4 acetylation and maintenance of the genome.

The *Arabidopsis* condensin II complex is also required for genome integrity and for pollen and embryo development (Schubert et al., 2013; Sakamoto et al., 2019; Municio et al., 2021). A combined spatial analysis of the localization of the 180-bp centromeric repeat and the 5S and 45S rDNA probes was performed in the *Arabidopsis cap-h2-2*, *cap-g2-1*, and *cap-d3-1* mutants (see Figure 5). Results indicated disassociation of the rDNA arrays from the centromeres, thus suggesting that direct binding of condensin II to rDNA array regions is necessary for their association with centromeres in *A. thaliana* (Sakamoto et al., 2019). However, cytological analysis of *Arabidopsis cap-d3* interphase nuclei indicated specific clustering of 45 rDNA sites, so CAP-D3 might localize in euchromatic loops and somehow determine the rigidity needed to maintain separation of chromocenters (Municio et al., 2021) In any case, an analysis of 3D chromatin organization by Hi-C may help clarify the distribution pattern of condensin II during interphase and its role in the clustering of rDNA arrays during interphase (Sakamoto et al., 2019). Evidence also indicates that SMC4/condensin is required for repression of a wide range of methylated genes subject to conditional expression (Wang et al., 2017). Gene examples are embryo and pollen vegetative cell-specific *GAMETE EXPRESSED-PROTEIN 1* (*GEX1, At5g5590*; Alandete-Saez et al., 2011), pollen tube-expressed *POLYMERASE DELTA 4* (*POLD4*, *At1g09815*; Wang et al., 2008), radiation-response factors such as *GAMMA-IRRADIATION AND MITOMYCIN C INDUCED 1* (*GMI1*, *At5g24280*; Böhmdorfer et al., 2011), and translesion-synthesis/homologous recombination factor *RECQ3* (*At4g35740*; Kobbe et al., 2009). Thus, breeding for condensin alleles may confer resistance to mitotic DNA damage caused by UVB and Al^3+^ and boost allele reshuffling *via* homologous recombination. Also because of the crucial role played by condensin in genome organization, these alleles may contribute to advance our knowledge in processes such as the regulation of MSUC and MSCI in crops.

## Opportunities For Engineering Modified Crossover Formation During Meiosis with Cohesins, Condensins, and Hormad Proteins

During meiosis, the reciprocal exchange of DNA between homologous chromosomes enables the reshuffling of parental genetic information and the transfer of the recombined material to the progeny (de Maagd et al., 2020). The formation of DSBs by the topoisomerase SPORULATION-DEFICIENT 11 (SPO11) is crucial for the initiation of synapsis between homologous chromosomes and CO formation (Esposito and Esposito, 1969; Ning et al., 2020). Then the MRX/N complex generates 3' single-strand DNAs at DSB sites that allow the recombinases RAD51 and DMC1 to bind to the single-strand DNA overhangs to promote search of homology and pairing. The subsequent simultaneous assembly of the multi-layered chromosome axis and synaptonemal complex by the α-kleisin REC8/SYN1, the coiled-coil proteins ASY3 and ASY4, the HORMA-domain protein ASY1 and transverse filament protein ZYP1 catalyze the successful synapsis of homologous chromosomes and the formation of COs (Ning et al., 2020). Thus, meiotic CO formation is an important target in crop breeding (de Maagd et al., 2020), especially because in modern crops, successive rounds of selection have reduced their genetic variation, leaving them with less allelic diversity than their wild relatives, called the “domestication bottleneck” (Dempewolf et al., 2017).

Meiosis starts with the formation of many DNA DSBs, most of which are repaired in non-CO events and do not result in recombinant chromosomes. In plants, only one to three DSBs per chromosome are processed into actual COs (de Maagd et al., 2020), and most of these COs exhibit interference, the phenomenon that prevents multiple COs from occurring in close proximity along each chromosome pair (Lambing et al., 2020b). In *Arabidopsis*, type I COs are sensitive to interference and are associated with the activity of the ZMM class of proteins, including mutS homolog 4 (MSH4), a meiotic-specific reciprocal-recombination factor. However, type II COs are insensitive to interference and are the product of the activity of MMS and UV sensitive 81 (MUS81), a restriction endonuclease (Taagen et al., 2020; Wang et al., 2020). At least three independent pathways suppressing CO formation have been identified in *Arabidopsis* (Taagen et al., 2020), featuring the activity of (1) RECQ4, a homolog of the human Bloom syndrome helicase (BLM) that forms the BTR complex along with TOP3α and RECQ MEDIATED INSTABILITY 1 (RMI1) and unwinds recombination intermediates to suppress class I COs; (2) FANCM, a DEAD/DEAH box RNA helicase, that is a direct DNA-binding cofactor along with MHF1 and MHF2, may unwind recombination intermediates, limits class II COs, and promotes synthesis-dependent strand annealing (SDSA); and finally (3) FIDGETIN-LIKE 1 (FIGL1), a Holliday junction resolvase, that forms a complex with FIDGETIN-LIKE 1 INTERACTING PROTEIN (FLIP), suppresses RAD51 and DMC1 recombination activity, and constrains strand invasion (Séguéla-Arnaud et al., 2017; Taagen et al., 2020).

*Arabidopsis* has a single *SCC2* homolog, and homozygous knockout alleles are embryonic-lethal. However, use of the non-lethal *Atscc2-5* allele revealed that during meiosis, *SCC2* participates in chromosomal axis formation, pairing of homologous chromosomes, synapsis, and recombination upstream of MSH4 and MUS81 (see Table 1). Genetic analyses also suggested that *SSC2* is epistatic to RAD51 during DSB formation, participates in the same pathway as *REC8*/*SYN1* and *WAPL1/2*, and is required for efficient CO resolution. Results from plant pull-down assays suggest that the N-terminus of AtSCC2 interacts with AtSCC4 *in vivo*, but knockdown of *AtSCC4* during meiosis does not cause any obvious meiotic defects, which suggests a divergence in the roles played by SCC2/SCC4 during mitosis and meiosis. For instance, in maize, *SCC4* is called *DEK15* (*Zm00001d052197*/*GRMZM2G079796*) and the corresponding *dek15/scc4* EMS-mutants do not show any obvious defects during meiosis. However, mitosis in endosperm and embryos is defective, with precocious sister chromatid separation, misaligned chromosomes, lagging chromosomes, and micronuclei. These mutants develop kernels with only 40% of the weight observed in the W22 reference line, with few and small starch grains in endosperm, and the embryos are arrested in development and die after 18 days (He et al., 2019). Notably, *DEK15* is required for proper expression of genes for the starch biosynthetic pathway, such as *Shrunken2*, *Starch synthase I* (*SSI*), and *SSIIa* (He et al., 2019), a finding of great agricultural importance.

The likely answer for the functional divergence between plant SCC2 and SCC4 may lie in the presence of a plant-specific homeodomain (with a C4-H-C3 amino acid motif) that is found only in SCC2, which is believed to mediate binding to histones and be required for meiotic function but not vegetative growth (Wang et al., 2020). Thus, in plants, only the activity of SCC2 is required for key meiotic processes such as CO resolution, a process of huge relevance for plant breeders.

Accurate and faithful transmission of chromosomes into the gametes relies on the establishment of COs between paternal and maternal homologous chromosomes during prophase I, just prior to first meiotic division, when COs, together with sister chromatid cohesion, guarantee proper chromosome orientation on the meiotic spindle. Meiotic chromosome modeling requires the assembly of axial elements that include meiosis-specific subunits of cohesin such as the meiotic α-kleisin REC8/SYN1 (Castellano-Pozo et al., 2020). In *Arabidopsis*, REC8/SYN1 is associated with regions of high nucleosome occupancy; histone methylation at H3K4 (expressed genes), H3K27 (silent genes), and H3K9 (silent transposons); as well as suppression of meiotic DSBs and COs (Lambing et al., 2020b). It may be a mechanism to guarantee that repeat-rich regions do not undergo meiotic recombination, in that these are highly susceptible to nonallelic COs that may threaten genome stability (Shahid, 2020).

In addition to its primary role in establishing sister chromatid cohesion, meiotic cohesin loading promotes the recruitment of HORMA-domain proteins (HORMADs) to the axial elements, so that chromosomes can initiate meiotic recombination *via* the formation of DNA DSBs and to undergo pairing of homologous chromosomes. Homolog pairing features the assembly of the synaptonemal complex (SC), a ladder-like structure that bridges the axial elements of aligned homologs. This process, known as synapsis, stabilizes homolog interactions and is essential to ensure that a subset of DSBs become CO-designated sites during the pachytene stage of meiotic prophase, which is defined by full synapsis. Thus, the processes leading to CO formation require the presence of meiosis-specific chromosome structures built over a cohesin scaffold (Castellano-Pozo et al., 2020).

In addition to the REC8/SYN1 α-kleisin, major components of the plant meiotic chromosome axis include the HORMAD protein ASY1 and the coiled-coil proteins ASY3 and ASY4 (Lambing et al., 2020a). In this primary configuration, coaligned chromatin loops project laterally from the axis, but at late prophase I, the axis is remodeled by the simultaneous depletion of HORMAD proteins and the loading of transverse filament SC proteins, including ZYP1a and ZYP1b (Lambing et al., 2020a). Recent work that combines chromatin immunolocalization techniques with tetrad analysis and immunostaining suggests that the *Arabidopsis* ASY1 protein is enriched in the centromeres, it antagonizes telomere-led recombination and it promotes spaced CO formation along the chromosomes *via* CO interference (see Table 1), during which a set of pro-CO factors act to protect interhomolog strand invasion events from antirecombination pathways (Lambing et al., 2020a). This finding hints at the possibility of reducing ASY1 activity to modify CO distribution in the genome and reduce linkage drag in major crops, which is major problem during the introgression of valuable alleles (Lambing et al., 2020a). For instance, results from modeling with R suggest that when a quantitative trait locus is within a region that is normally CO-poor, boosting the recombination rate may decrease the linkage drag nearly 10-fold after the foreground selection and may improve the return to the recurrent parent (Falque and Martin, 2020). In *Arabidopsis*, high temperature during meiosis (36–38°C, 24 h) reduces SPO11-dependent DSB formation, as observed by labeling with γ-H2AX, formation of DMC1 foci, recruitment of ASY4 to the chromosome axis during zygotene, and recruitment of ASY1 to the SC (during zygotene as well). Although heat treatment does not affect the distribution and abundance of REC8/SYN1 in chromosomes during zygotene and pachytene (Ning et al., 2020), loading of ASY1 is not defective in *syn* mutants exposed to heat, which suggests that the presence of REC8/SYN1 disturbs the proper loading of ASY1 during high temperatures (Ning et al., 2020). The biochemical nature of this interaction during heat has not been determined, but if it were optimized, it might allow the adaptation of crops to high temperatures.

## Efficient Transmission of New Alleles: Boosting CO Formation with Meiotic Cohesins

During meiosis in mouse oocytes, mutual recombination between parental homologous chromosomes creates chiasmata, the physical linkages that bind bivalents during meiosis I (Silva et al., 2020). Cell cycle-dependent cleavage of the meiotic α-kleisin protein REC8/SYN1 that is present on the chromosome arms allows segregation of homologous chromosomes during anaphase I. Then during meiosis II, proteolytic cleavage of REC8/SYN1 at the centromeres by the Cys-protease separase (ESP1) allows centromeres to segregate from one another in the metaphase to anaphase transition (see Table 1; Yang et al., 2009, 2019; Hsieh et al., 2020). In general, the meiotic cohesin complex may regulate recombination and topology. In comparison to the mitotic α-kleisin SCC1, loading of meiotic α-kleisin REC8/SYN1 leads to early firing of replication origins and fast progression of replication forks, perhaps as a mechanism to facilitate passage from the G1 to the S phase (Hsieh et al., 2020). These results suggest that directed evolution in plant α-kleisins might optimize replication during meiosis, and perhaps may lead to the discovery of (a) heat-tolerant REC8/SYN1 alleles better able to load ASY1 or (b) discovery of useful, weak or loss-of function *REC8*/*SYN1* alleles. For instance, in *Kitaake* rice, an *rec8* allele is combined with the alleles *osd1* (*OSD1*, *OMISSION OF SECOND DIVISION*; regulation of cell cycle progression) and *pair1* (*PAIR1*, *HOMOLOGOUS PAIRING ABERRATION IN RICE MEIOSIS1*, control of DSB formation) to induce the apomictic-like and commercially valuable phenotype known as *Mitosis instead of Meiosis* (*MiMe*, *Osrec8*/*Ososd1*/*Ospair1*; Khanday et al., 2019). In this way, hybrid vigor (e.g., heterosis) can be fixed and maintained in the genotype of clonal progenies (Wang, 2020).

Cohesin is actively released from DNA by the separase protease (Silva et al., 2020), which targets the α-kleisin REC8/SYN1 (Faramarz et al., 2020). In yeast, before the metaphase-to-anaphase transition, the securin protein (the corresponding *Arabidopsis* orthologs are PATRONUS1/2) inhibits the protease activity of the separase. Then at the onset of anaphase, securin is itself degraded by the anaphase-promoting complex/cyclosome (APC/C), thus freeing the separase (Yang et al., 2009; Cromer et al., 2019). Cleavage of the REC8/SYN1 α-kleisin along chromosome arms at anaphase I by the separase allows for resolution of chiasmata, formed as a result of homologous chromosome recombination and the faithful segregation of homologous chromosomes (Yang et al., 2009; Silva et al., 2020). However, centromeric cohesion is protected by the conserved SUGOSHIN (SGO1/2 in *Arabidopsis*) family of proteins until anaphase II, when separase-mediated cleavage of REC8/SYN1 facilitates the separation of sister chromatids (Yang et al., 2009; Cromer et al., 2013). In *Arabidopsis*, the AESP1 separase regulates a wide range of important developmental processes such as meiotic chromosome segregation, tetrad formation, embryo development up to the globular stage, cellularization of the endosperm, proper assembly of the radial microtubule system after telophase II, and even N-acetylation of proteins required for vesicle trafficking such as the H^+^ and V-ATPases (Yang et al., 2009; Liu et al., 2017b; see Table 1). However, separase homologs have not been characterized in major crops, and this gap may be limiting our understanding of chiasmata resolution, among many other processes of great agricultural relevance.

In eukaryotic organisms such as budding yeast and *Arabidopsis*, ample evidence indicates that WAPL is involved in the release of cohesin from meiotic chromosomes and its activity is essential for normal meiosis, especially for the release of REC8/SYN1 during prophase I (De et al., 2016; Silva et al., 2020). In mouse oocytes, deletion of *WAPL* leads to the formation of aberrant chiasmata-like structures, not because of an increase in CO formation but because of an increase in DNA damage, as observed by an increase in γ-H2AX foci, presumably linked to retention of the kleisin and increased rigidity, leading to inefficient homologous repair and formation of chromosome bridges. Structural analysis by Hi-C revealed that loss of *WAPL* during meiosis also leads to the formation of chromatin loops within loops (Silva et al., 2020), a phenotype not yet reported in *Arabidopsis*. Formation of aberrant chiasmata has not been documented in *Arabidopsis wapl* mutants either.

Perhaps we must consider how cohesin is loaded onto chromatin in the first place. Work in budding yeast suggests that during the G1 phase, proper loading of cohesin onto chromosomes depends on the remodeling structure of chromatin (RSC) chromatin remodeler, a member of the SWI/SNF family of ATPases (Tsukiyama et al., 1999; Muñoz et al., 2020). The RSC complex consists of 19 subunits, is enriched in centromeres, and is believed to function as a chromatin receptor that allows direct recruitment of SCC2/SCC4 on broad nucleosome-free-regions, which are probably remodeled by RSC *via* nucleosome eviction or DNA translocation. These nucleosome-free regions usually correspond to promoters where the cohesin loader is normally found (Muñoz et al., 2020). Other chromatin remodelers known to act as cohesin loaders in humans such as Imitation Switch 1 (ISW1) and Chromo-ATPase/helicase-DNA-binding domain (CHD1) support cell proliferation, so the SCC2/SCC4 module may be somehow promiscuous and may become functional in the proximity of alternative chromatin remodelers during cohesin-dependent processes such as DNA repair (Stokes et al., 1996; Tsukiyama et al., 1999; Muñoz et al., 2020; see Table 1). Further research could determine whether alternative meiotic chromatin remodelers exist in plants and identify the corresponding phenotypes.

In *Arabidopsis*, thorough work has helped uncover a meiotic antagonist of WAPL that may operate during prophase I by binding to PDS5 and displacing PDS5 from WAPL. This antagonist is the SWITCH1/DYAD protein (SWI1), a protein of unknown biochemical function and whose actual *modus operandi* is also ignored. The phenotype of the *swi-1* mutant features failure to assemble the chromosome axis and to establish sister chromatid cohesion. The biochemical evidence (GST pull-down assay in *Escherichia coli* and bimolecular fluorescence complementation in tobacco) indicates that SWI1 strongly interacts with the N-terminus of PDS5A, specifically the N-terminal 300 amino acids, but is also able to interact strongly with PDS5C/E and mildly with PDS5B/D, that is, all PDS5 proteins in *Arabidopsis*. WAPL was also found to bind to the N-terminus of PDS5A, which suggests the existence of a potentially antagonistic entanglement that was confirmed by a competitive binding assay in tobacco leaf cells, in which WAPL was displaced from PDS5 by SWI1. The APC is believed to target SWI1 for degradation from zygotene onward, as suggested by prolonged chromatin residency and reduced fertility in *SWI1* mutants with mutation of five tentative D-boxes. Of note, in the *rec8* mutants, the SWI1-GFP signal is retained, which suggests that SWI1 may interact with yet unidentified meiotic cohesin complexes with alternative α-kleisins and that *swi1* mutants might show an increase in the formation of multiple female meiocytes, thus suggesting a role in the specification of meiocyte identity (see Table 1; Yang et al., 2019). The *Arabidopsis SWITCH1/DYAD* gene has homologs in rice and maize, called *AMEIOTIC1* (Pawlowski et al., 2009; Che et al., 2011; Yang et al., 2019), which indicates the possibility of validating these phenotypes in these crops.

## Conclusion

**Figure 6:**
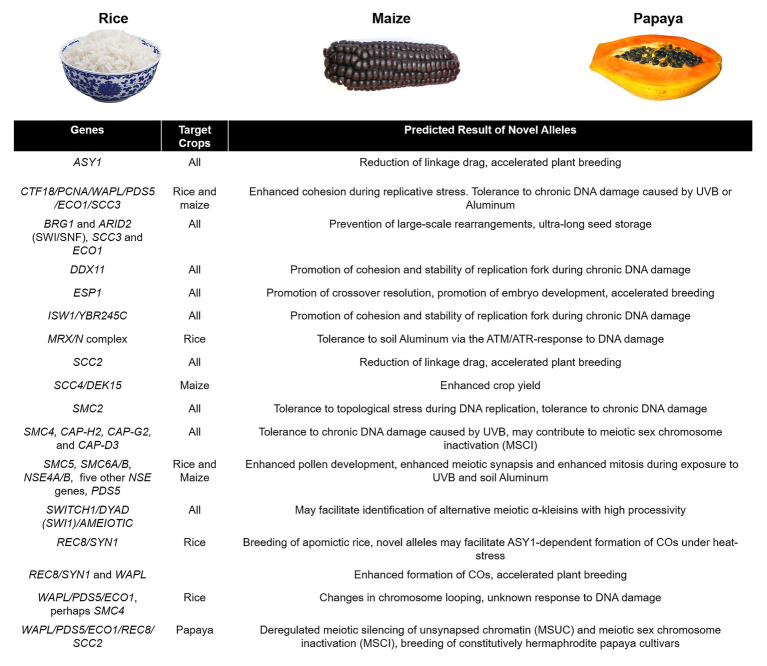
Suggested agricultural targeting of genes involved in SMC-regulated processes. In major crops such as rice and maize, it might be possible to stabilize transcription and homologous recombination under chronic stress caused by UVB radiation and soil aluminum, and in the case of papaya, it might be possible to manipulate sex chromosome silencing to breed all hermaphrodite progenies. Illustrative images were obtained from Google Images and Wikipedia (CC BY-SA 2.0 and CC BY-SA 4.0).

Biomedical research in yeast and mammalian cohesin and condensin complexes and their respective regulators has contributed significantly to our understanding of the processes they affect, namely genome maintenance, meiotic recombination, CO formation, and regulation of high-order chromatin structure in chromosomes. Research in the model plant *A. thaliana* and crops has also pushed the boundaries of our knowledge and facilitated charting the way to applied plant breeding by providing clear mutant phenotypes and solid genetic evidence (de Maagd et al., 2020). Translation of basic research into crop breeding may speed the screening and retrieval of valuable alleles for tolerance to DNA damage by UVB and aluminum exposure (for a summary see Figure 6). It may help in manipulating CO frequency and distribution for enhanced transmission of key traits, both wild and mutant, at a time when climate change and population growth threaten food security (Gerland et al., 2014; Zhao et al., 2017; Lambing et al., 2020b; see Figure 6). Efficient genome-editing methods by CRISP-Cas9 have been developed for crops (Qi, 2019), so we may finally be able to perform detailed genetic, cytological, and biochemical characterization of SMCs in maize and rice. In conclusion, a better understanding of the establishment of cohesion and genome organization in crops may contribute to increases in yield under less-than-ideal environmental conditions.

## Author Contributions

The author confirms being the sole contributor of this work and has approved it for publication.

### Conflict of Interest

The author declares that the research was conducted in the absence of any commercial or financial relationships that could be construed as a potential conflict of interest.
